# Genome sequences of human anelloviruses in the *Lamedtorquevirus*, *Memtorquevirus*, and *Samektorquevirus* genera identified from the female genital tract

**DOI:** 10.1128/mra.00582-24

**Published:** 2024-08-05

**Authors:** Eric D. Do, Steven C. Holland, Emily A. Kaelin, Caroline Mitchell, Jaime Soria, Alberto La Rosa, Eduardo Ticona, Robert W. Coombs, Lisa M. Frenkel, Marta E. Bull, Efrem S. Lim

**Affiliations:** 1 School of Life Sciences, Arizona State University, Tempe, Arizona, USA; 2 Center for Fundamental and Applied Microbiomics, the Biodesign Institute, Tempe, Arizona, USA; 3 Department of Obstretrics and Gynecology, University of Washington, Seattle, Washington, USA; 4 Infectious Diseases Departmento, Hospital Nacional Dos de Mayo, Universidad de San Marcos, Lima, Peru; 5 Asociaciòn Civil Impacta Salud y Educación, Lima, Peru; 6 MSD Peru, Lima, Peru; 7 Infectious Diseases Departamento, Hospital Nacional Dos de Mayo, Asociaciòn Civil Impacta Salud y Educación, Universidad de San Marcos, Lima, Peru; 8 Departments of Medicine; Laboratory Medicine and Pathology, University of Washington, Seattle, Washington, USA; 9 Seattle Children’s Research Institute, Seattle, Washington, USA; 10 Department of Pediatrics, University of Washington, Seattle, Washington, USA; DOE Joint Genome Institute, Berkeley, California, USA

**Keywords:** *Anelloviridae*, female genital tract

## Abstract

We identified and characterized seven anellovirus genome sequences in the female genital tract through virome metagenomic sequencing of cervicovaginal lavage specimens from women living with HIV in Peru. Phylogenetic and genomic analyses indicate that they belong to three newly proposed *Lamedtorquevirus*, *Memtorquevirus*, and *Samektorquevirus* genera in the *Anelloviridae* family.

## ANNOUNCEMENT

Anelloviruses (family *Anelloviridae*) are small, nonenveloped viruses with a circular negative sense, single-stranded DNA genomes typically ranging between 1.6 and 3.9 kb in length (1). In humans, anelloviruses can be found in a broad range of body sites including blood, stool, and the female genital tract (2–4). While anelloviruses are not known to be associated with disease, changes in anellovirus load have been implicated in host immune competency such as organ transplant immunosuppression (5).

**Fig 1:**
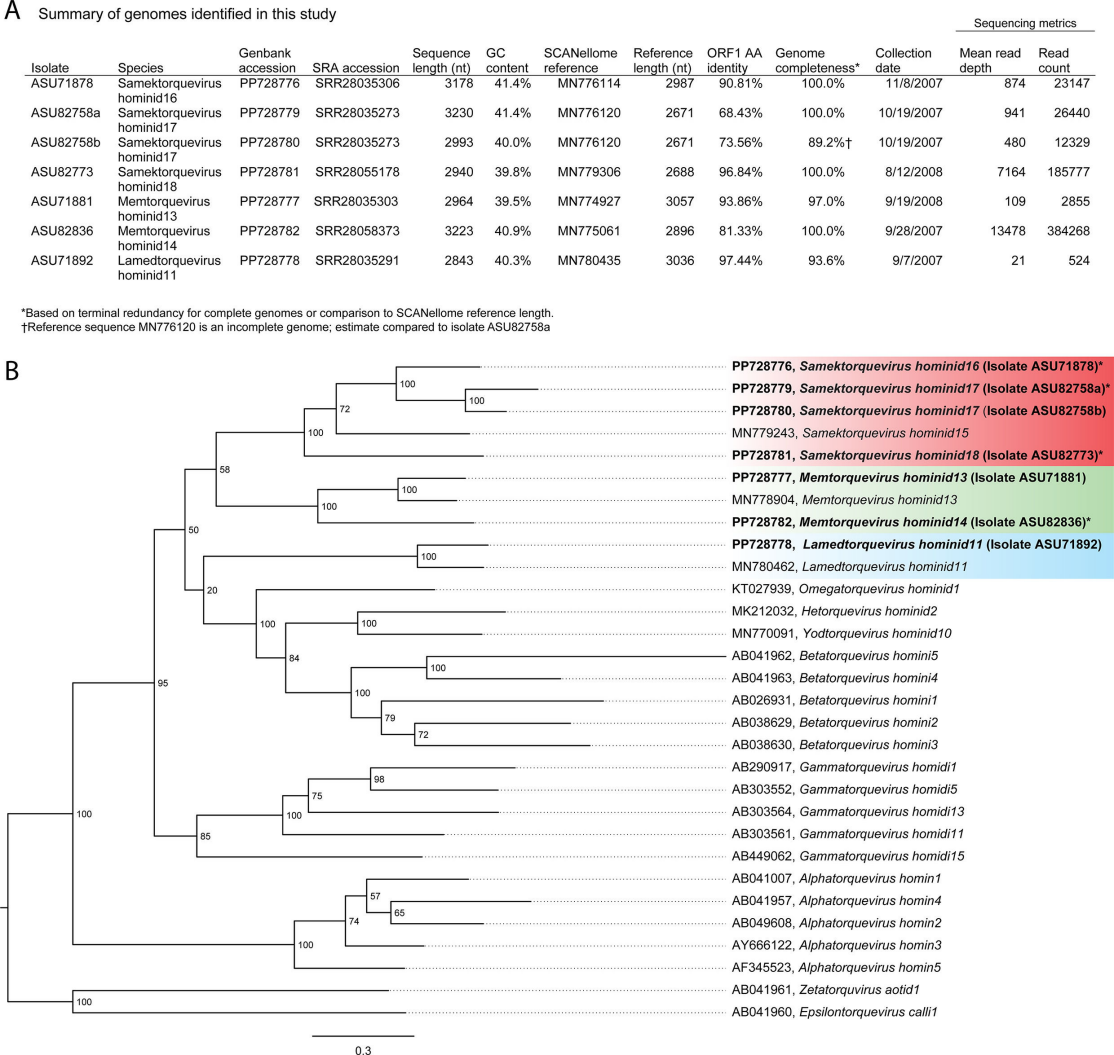
(**A**) Summary details of genome sequences. (**B**) Phylogeny of anellovirus ORF1 amino acid sequences. Sequences identified in this study are in boldface, with complete genomes denoted by an asterisk. Genbank accession numbers and virus species names are indicated. Bootstrap support values are labeled at tree nodes.

As part of ongoing efforts to characterize the human virome, we conducted virome metagenomic sequencing of 31 women living with HIV over a 2-year period (125 cervicovaginal specimens) in Lima, Peru who were on long-term antiretroviral therapy (6, 7). Cervicovaginal lavage specimens were subjected to virus-like particle enrichment (filtered through 0.2 µm pore filter, digestion of non-encapsidated nucleic acids with benzonase and Baseline-ZERO DNase), and total nucleic acid extraction (bioMérieux eMAG). Viral DNA was amplified using multiple displacement amplification (GenomiPhi V2), used for library preparation (Illumina DNA Prep library kit), followed by next-generation sequencing (Illumina NextSeq 2000 2 × 150 bp paired-end reads). Sequencing reads were trimmed to remove adapter sequences [Cutadapt v.4.0 (8)], quality filtered (trimq = 30, min length = 75, min avg quality = 20), PhiX and human host read removed, and deduplicated (BBTools). Contigs were assembled with metaSPAdes [V. 3.15.4 (9)] and queried against viral protein sequences from NCBI RefSeq and viral neighbor genome sequences (downloaded on January 2023) (10) using blastx (e-value 1 × 10^−3^). All tools were run with default parameters unless specified. We identified seven contig sequences collected from six individuals from September 2007 through September 2008 that had limited identity to *alphatorquevirus*, *betatorquevirus*, and *gammatorquevirus* sequences that are typically the most prevalent human anelloviruses. Based on terminal redundancy, four sequences were circular, full-length, complete genomes, and three were near-complete genomes (Fig. 1A).

The International Committee on Taxonomy of Viruses and community guidelines for genus demarcation criteria are based on the ORF1 amino acid sequences (1, 11). We performed a phylogenetic analysis on the ORF1 amino acid sequence alignment using MAFFT v. 7.505 (12), trimmed with trimAl (v.1.4.rev15) using the -gt 0.2 gappyout option (13) of our seven genome sequences and 23 reference sequences from the *Samektorquevirus*, *Memtorquevirus*, *Lamedtorquevirus*, *Alphatorquevirus*, *Betatorquevirus*, *Gammatorquevirus*, *Hetorquevirus*, *Omegatorquevirus*, *Yodtorquevirus*, *Epsilontorquevirus*, and *Zetatorquevirus* genera. The maximum likelihood phylogeny was constructed with IQ-TREE (v. 2.0.3) with the model VT + F + G and 1,000 bootstrap replicates (14) and visualized in FigTree (v. 1.4.4) (15). The seven new sequences grouped with members of the newly proposed *Lamedtorquevirus*, *Memtorquevirus*, and *Samektorquevirus* genera (16), designated as Lamedtorquevirus hominid11 (GenBank accession number PP728778), Memtorquevirus hominid13 (PP728777), Memtorquevirus hominid14 (PP728782), Samektorquevirus hominid16 (PP728776), Samektorquevirus hominid17 (PP728779, PP728780), and Samektorquevirus hominid18 (PP728781) (Fig. 1B).

These findings highlight the understudied nature of the female genital tract virome and demonstrate the inclusion of human anelloviruses in the *Lamedtorquevirus*, *Memtorquevirus*, and *Samektorquevirus* genera within the *Anelloviridae* family.

All study participants provided informed consent. This study was approved by the Institutional Review Boards of the Hospital Dos de Mayo (Lima, Peru), Seattle Children’s Hospital (Seattle, Washington, USA), and Arizona State University (Tempe, Arizona, USA).

## Data Availability

The sequences of the seven genomes of anellovirus identified in this study have been deposited into the NCBI GenBank database under accession numbers: PP728776–PP728782. Raw sequencing reads have been deposited into the NCBI SRA under project number PRJNA1077994.
